# Eventual Convergence of the Reputation-Based Algorithm in IoT Sensor Networks

**DOI:** 10.3390/s21186211

**Published:** 2021-09-16

**Authors:** Jacek Lebiedź, Piotr Cofta, Cezary Orłowski

**Affiliations:** 1 Faculty of ETI, Gdansk University of Technology, 80-233 Gdańsk, Poland; 2 Institute of Management and Finance, WSB University in Gdansk, 80-266 Gdańsk, Poland; pcofta@wsb.gda.pl (P.C.); corlowski@wsb.gda.pl (C.O.)

**Keywords:** IoT sensor networks, EWMA algorithms, eventual convergence, proof of convergence

## Abstract

Uncertainty in dense heterogeneous IoT sensor networks can be decreased by applying reputation-inspired algorithms, such as the EWMA (Exponentially Weighted Moving Average) algorithm, which is widely used in social networks. Despite its popularity, the eventual convergence of this algorithm for the purpose of IoT networks has not been widely studied, and results of simulations are often taken in lieu of the more rigorous proof. Therefore the question remains, whether under stable conditions, in realistic situations found in IoT networks, this algorithm indeed converges. This paper demonstrates proof of the eventual convergence of the EWMA algorithm. The proof consists of two steps: it models the sensor network as the UOG (Uniform Opinion Graph) that enables the analytical approach to the problem, and then offers the mathematical proof of eventual convergence, using formalizations identified in the previous step. The paper demonstrates that the EWMA algorithm converges under all realistic conditions.

## 1. Introduction

Let us consider a dense Internet of Things network, consisting mostly of inexpensive sensors that collectively measure a certain physical phenomenon. By design, this network may comprise some high-quality nodes (characterized, among others, by the low level of uncertainty) as well as a number of nodes of high or unknown uncertainty. In order to improve the overall quality of its measurement, the network may want to learn about the quality of its nodes to decrease the impact of the low-quality ones. In the absence of external reference sources, the network must rely on opinions randomly issued by one node about the other to be converted into a reputation of those nodes. Note that the notion of quality applies equally well to nodes that provide incorrect readings due to the use of low-quality sensors and nodes that provide such readings due to their failure or the failure of their communication links. This makes this approach potentially attractive to IoT networks that use wireless communication.

One of the popular algorithms to perform such conversion is the EWMA one (Exponentially Weighted Moving Average), which is widely used, e.g., in social networks. The algorithm tends to perform well under simulated conditions, but, to the author’s knowledge, there is no proof that it eventually converges, i.e., that the algorithm eventually delivers the stable value (or, e.g., asymptotically approaching the stable value) of the reputation of the node, provided that the network itself is in a stable situation. Undesired behavior of the algorithm may include oscillations (where a stable value is only momentarily achieved), or false assessment (where the algorithm gradually diverges from the assessment), or the failure to provide the assessment (where the outcome of the algorithm becomes unrealistic).

The key contribution of this paper is proof of the eventual convergence of the EWMA algorithm, as applied to IoT sensor networks. The proof is also applicable to other uses of the EWMA, e.g., in social networks, provided that similar conditions are met. The approach presented here can also be applicable in related areas where the EWMA or its derivatives are used, e.g., in combating fake news.

The proof presented here consists of two steps. In the first step, the formal model of the sensor network is introduced. The model, described here as the UOG (Uniform Opinion Graph), enables the analytical approach to the problem of convergence, thus allowing for a deeper analysis than the usual simulation. The UOG also enables the second step of the proof, where the mathematical proof of eventual convergence is provided. The paper demonstrates that the EWMA algorithm converges under all realistic conditions.

This paper starts with a brief literature review that introduces the subject of research. The formalization of the EWMA algorithm, as used in IoT sensor networks, follows. Then the UOG is introduced, and its use is justified to demonstrate that the UOG is a suitable representation of the EWMA for random graphs. The preliminary analysis of the convergence of the EWMA for the UOG is then conducted. From there, the mathematical proof follows. Conclusions close the paper.

## 2. Literature Review

This literature review is structured as follows. First, it provides certain background into the use of reputation in IoT sensor networks, with a special focus on the use of the EWMA algorithm. Next, it discusses papers that are directly relevant to the convergence of the formula derived from the EWMA algorithm.

The use of reputation and trust has its sources in social networks [1]. However, once networks are designed so that nodes can provide opinions about other nodes, the same concept, as well as the associated algorithms, can also be applied to IoT sensor networks (see the work of [2] for the distinction between sensor networks and IoT sensor networks). Such types of networks increasingly gain popularity, e.g., in sensing air pollution [3]. For a more general overview of technology and applications, see, e.g., the work of [4] or [5]. Considering existing literature, currently, the important focus seems to be the identification of faulty or malicious nodes (e.g., using reputation scoring [6]), i.e., nodes that unintentionally or intentionally deliver incorrect readings. This calls for some form of trust management that allows for identifying and acting on nodes deemed untrustworthy [7]. Various proposed trust models (i.e., reputation-scoring algorithms) are applied to IoT networks. For example, the work of [8] lists no less than 80 algorithms in use. Those algorithms employ various paradigms, be it various versions of averaging, fuzzy logic [9], or neural networks [10].

When it comes to reputation, algorithms that employ averaging, specifically the EWMA one, are among the most popular ones. They demonstrate their robustness while dealing with opinions provided by humans [11]. They have also been used widely to calculate the reputation of automated services [12], as well as in other applications, e.g., to monitor changes in the mean value of the process [13] and in forecasting [14], to name but a few areas. In [15], the applicability of EWMA to the calculation of reputation in dense sensor networks was demonstrated to decrease the uncertainty of data produced by such networks. Note that the EWMA algorithm exists in many variants, namely SMA (Simple Moving Average), EWMA, PEWMA (Probabilistic EWMA) [16], AEWMA (Adaptative EWMA) [17], etc. It is also worth noting that the EWMA algorithm discussed here is similar yet differs in its application in comparison with EWMA smoothing [18], the latter being the variant of exponential smoothing.

The performance, including the convergence of such an algorithm, is a going concern. However, the primary interest seems to be the optimization of the parameters of the algorithm, specifically at its initial phase, where, e.g., the maximum entropy principle can be applied [19]. Regarding the convergence of EWMA, it is assumed and accepted that the algorithm delivers eventual convergence when used in control or data smoothing applications, i.e., in situations where the outcome of the algorithm does not directly affect its input. In such situations, the noise seems to be the primary concern, and the potential for the convergence despite the noise was analyzed, e.g., in the work of [20], using the Markov Chain.

EWMA is equally popular in social networks and in other applications that collate opinions into reputation [21]. This application may be different, as the reputation-scoring algorithms tend to have the inner feedback loop where the nodes of higher reputation are able to affect the scoring of other nodes. Further, the operation of the algorithm over the random graph of opinions differs from its operation in control or data smoothing. Despite its popular use, the authors were unable to find any proof of the eventual convergence of the EWMA algorithm used for the assessment of reputation in sensor (or social) networks, i.e., the proof that under stable conditions, the algorithm eventually converges to the stable assessment of the situation. Instead, simulations under various, often variable conditions, as well as the use of real data sets, are widely used, using available toolkits [22].

This paper proposes that the convergence of the EWMA algorithm in random graphs can be analyzed as a special case of the convergence of linear fractional transformations (homographic functions). The convergence of such functions is known to strongly depend on their parameters. The problem of convergence has been studied from mathematical perspectives in a way that is not always completely relevant to the problem at hand. Differences tend to focus on the range of parameters that are covered by the literature.

Hillam and Thorn [23] demonstrated the convergence of homographic functions over complex support. However, the range of parameters that have been studied does not satisfy the use case presented in this paper, thus limiting the use of the proof demonstrated in the work of [23] only to special cases of only some practical uses.

Mandell and Magnus [24] provide an interesting classification of homographic functions (over the complex support) depending on the properties of their normalized matrices. Four types of functions are analyzed: parabolic, elliptic, hyperbolic, and loxodromic, and conditions for their convergence are identified. The proof demonstrated in this paper significantly improves on the work of [24], as it not only demonstrates convergence but also demonstrates convergence within the range specified by parameters, thus assuring that the result of such convergence always satisfies the use case.

Note, however, that the results presented in the work of [24] do not contradict the results presented here. For particular combinations of parameters and for some stages of the proof, they lead to the same results.

## 3. The EWMA Algorithm in IoT Networks

The use of the EWMA algorithm to decrease the uncertainty in IoT sensor networks has been introduced in the work of [15]. It is characteristic for the IoT networks, in contrast with professional measurement networks, that they may contain sensors of varied quality and that the maintenance protocols, as well as communication protocols, may not be up to high standards. Consequently, measurement results obtained from such networks tend to bear higher uncertainty, so that the ability to contain this uncertainty is important.

Reference nodes (i.e., nodes of assured high quality) are often used to contain the growth of uncertainty by acting two ways: to provide the set of quality readings and also to judge other nodes and eliminating those that seem to be faulty. However, those solutions are both expensive in operation and sensitive to the failure of one of the reference nodes.

The approach proposed in the work of [15] disposes of the need for reference nodes, but it requires nodes to be over-provisioned. Considering that the cost of the lower-quality node can be significantly lower than the cost of the reference node, such a trade-off can be quite attractive. This approach is based on the ability to tell sensors of low uncertainty from those of high uncertainty, so the high uncertainty of some of them does not overly taint the measurements performed by the network. As the network is autonomous, it cannot use external sources or reference nodes to perform the assessment of nodes to learn about their uncertainty. Instead, it must rely on opinions issued by nodes within the network about each other.

Those opinions are issued by nodes on the basis of their assessment of whether other nodes perform as expected. As the node can issue the opinion only based on its own performance, high-quality nodes tend to issue correct opinions while low-quality nodes tend to issue incorrect ones. What the opinions contain is traditionally called the level of trust, by analogy to subjective trust [1] used in social networks.

The network can employ the algorithm that computes those opinions into the reputation of each node, where the weight of opinion of the node depends on its current reputation at the time the opinion has been issued. That is, the higher the reputation of the node, the more influential its opinion is. One of the formalizations of this algorithm is known as the EWMA, and this is the one analyzed here. This formalization combines weighted averaging of opinions with their decay so that older opinions weigh less toward the current reputation. This formalization, as used through this paper, can be expressed as

(1) $$w_{a}^{\tau }=\frac{\sum_{i=1}^{n}\left(u_{b\to a}^{\tau_{i}}\cdot conf_{b}^{\tau_{i}}\right)}{\sum_{i=1}^{n}conf_{b}^{\tau_{i}}}$$

where:

- $w_{a}^{\tau }$ reputation of the sensor *a* at the moment *τ*
- *n* number of opinions about *a* that are known to the algorithm and that were provided before or at the time *τ*
- $u_{b\to a}^{\tau_{i}}$ level of trust that the sensor b, providing the *i*-th opinion, had in sensor *a* at the time the opinion has been provided, *τ_i_*.
- $conf_{b}^{\tau_{i}}$ confidence in the *i*-th opinion, being the confidence in the sensor *b* that provided this opinion, calculated for the time the opinion has been provided, $\tau_{i}$. This confidence is calculated as $conf_{b}^{\tau_{i}}=c_{b\to b}^{\tau_{i}}\cdot w_{b}^{\tau_{i}}\cdot f_{d}\left(\tau ,\tau_{i}\right)$ where:
  - $c_{b\to b}^{\tau_{i}}$ self-confidence of the sensor *b* that provided the *i*-th opinion in its own judgment at the time the opinion has been issued (i.e., $\tau_{i}$); this self-confidence is used when calculations made by the sensor include elements of doubt.
  - $w_{b}^{\tau_{i}}$ reputation of the sensor *b* that provided the *i*-th opinion at the time the opinions has been issued, $\tau_{i}$
  - $f_{d}\left(\tau ,\tau_{i}\right)\in \left[0\ldots 1\right]$ the decay function that decreases the influence of opinions that are less current; the function takes as its parameters the time moment τ when the calculation was performed and time moment $\tau_{i}$ when the *i*-th opinion was issued; for EWMA, the exponential decay function is used so that $f_{d}=e^{-\lambda \left(\tau -\tau_{i}\right)}$ where λ is the decay constant.

There are few families of algorithms that can be used to establish what, in fact, is a reputation of the node, out of opinions about such a node, coming from unreliable peers [12]. The EWMA algorithm is a prominent example of the deterministic approach, where the resulting reputation bears no uncertainty. Alternatively, the Bayesian approach can be used (e.g., the work of [25]) so that the reputation of the node can bear its own measure of uncertainty. Fuzzy systems are also used to better reflect the human perception of reputation (e.g., the work of [26]). Machine learning (where examples of various quality are presented to the algorithm) is used as well [27], but usually in the context of determining malicious nodes, not just low-quality ones.

The EWMA is one of the simplest deterministic algorithms, where only direct (first-level) opinions are considered, i.e., opinions resulting from the direct observation of one node by others or from observing the same phenomenon. More complex algorithms may take into account also second- and third-level opinions, i.e., opinions about opinions provided by nodes. The choice of this was dictated by the desire to avoid the second-order considerations about the uncertainty (i.e., the uncertainty introduced by the algorithm itself), while neither the similarity to human reasoning nor the ability to learn from examples was particularly important.

Further, as the research interest was in demonstrating eventual convergence, the EWMA algorithm forms the natural starting point, being both less complicated and better understood than other algorithms.

The decrease in uncertainty provided by the use of the EWMA algorithm is contingent on the IoT network being reasonably “dense”, i.e., on situations where the neighbors of the given node can-at least from time to time-provide opinions about the given node. The underlying assumption is that the network is over-provisioned, i.e., that there are significantly more sensors in the network than would have been normally used. This concept is explained in detail in the work of [15]. It is worth mentioning that examples provided throughout this paper tend to use the over-provisioning of about four, thus assuming a grid-like structure of sensors. Such a level of over-provisioning can be reasonably achieved in networks that measure physical phenomena such as air pollution. Additional costs can be compensated by using lower-quality sensors.

Note that the research presented in this paper demonstrates that the convergence itself is not contingent on any particular level of over-provisioning, but the speed of such convergence is. The convergence is only contingent on opinions being provided, i.e., on the ability of one node to assess the other node.

Modern implementations of IoT networks may rely on wireless multi-hop communication (e.g., mesh networks [28]) to deliver the readings from the sensors to the central processing platform. Such networks may employ their own routing algorithms to optimize their operation and to eliminate misbehaving nodes, e.g., trust-based routing, using their own algorithms. The EWMA algorithm described here neither suffer from nor leverage such solutions. It assumes that the underlying communication network eventually delivers results but requires neither timeliness nor reliability. It is generally immune to delays and incidental omissions, including the disappearance and re-appearance of nodes. Further, it is intended to be implemented at the central processing platform so that it does not increase the workload on the network or on the nodes themselves.

Note that this paper assumes that all the nodes are benevolent. This paper does not study the convergence under adversary attacks where, e.g., malicious nodes are added to the network or benevolent ones became malicious.

## 4. The Uniform Opinion Graph (UOG)

The realistic model of the IoT sensor network is a random graph, with nodes representing sensors and edges representing opinions. While nodes remain unchanged, edges are created and disappear randomly in time, as new opinions are created, and old ones are discarded.

The interest of this paper focuses on a directed random graph with a specific average density. Such a graph is a variant of the Erdos-Renyi or Gilbert graphs. It is known that the probability distribution of the degree of the node follows the binomial distribution and reaches its maximum for the degree being equal to twice the average density, evenly split between inbound and outbound edges. If there is a fixed proportion of nodes of specific colors (types), this proportion is also reflected in the distribution of inbound and outbound edges.

**Figure 1:**
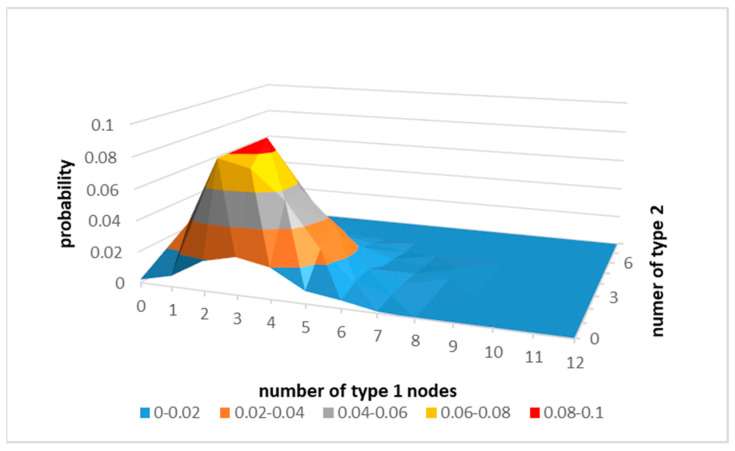
The simulated probability density distribution of antecedents of the node as a function of the number of “type 1” and “type 2” nodes in random graphs.

The number and the distribution of antecedents are of interest in this paper. For example, using simulated results (see Figure 1.) for random graphs of 20 nodes that have only two types of nodes: 12 nodes of type 1 and 8 nodes of type 2, with the average density of 5, the dominant configuration of a node is that the node has five antecedents of which three are of type 1, and two are of type 2.

Therefore, for the purpose of the analysis presented here, the simple model of a random graph is used that assumes that every node of the graph has exactly the same configuration that follows the dominant one. This model is called the UOG (Uniform Opinion Graph).

- Taking this model further, one can assume that there is a limited set of types of nodes (called further “roles”), each type representing a node of a certain quality. Assuming the uniform and fixed distribution of opinions within the UOG, it is enough to analyze only as many nodes that there are roles, each node being a faithful representative of all nodes with the same role. Further simplifications lead to the UOG graph, whose properties are suitable for mathematical analysis;
- There are only two roles: trustworthy nodes and faulty ones. Trustworthy nodes are of high quality, and faulty nodes are of low quality. Note that the phrase “faulty” does not imply that they failed in any way but that their quality is significantly inferior to those considered trustworthy;
- The complete UOG is defined by three parameters: *p*-number of trustworthy nodes that provide an opinion about the given one; *q*-number of faulty nodes that provide an opinion, and a *multiplier* that allows constructing graphs of different sizes;
- Roles of nodes are fixed; there are *p* · *multiplier* trustworthy nodes and *q* · *multiplier* faulty ones; neither those roles nor their allocation to nodes change, allowing for stable conditions;
- The graph density is fixed to p+q; each node receives opinions exactly from *p* trustworthy and *q* faulty nodes;
- Opinions can carry only one of two values: *t* or *f*; those values represent the value $u_{b\to a}^{\tau_{i}}$. from the same Equation (1). The value *t* is reported by a node about the node of the same role (i.e., trustworthy about trustworthy, faulty about faulty); *f* is reported otherwise;
- Self-confidence of any node is set to 1, i.e., from Equation (1); $c_{b\to b}^{\tau_{i}}=1$;
- Initial reputation of the node is the same for all nodes, set mid-point between *t* and *f;*
- The analysis is performed in steps, identified by the index *i*, where *i* identifies the current assessment, *i −* 1 the previous one, and so on;
- In each step, the analysis calculates the new reputation for the representative trustworthy node (value *x*) and for the representative faulty node (value *y*).

**Figure 2:**
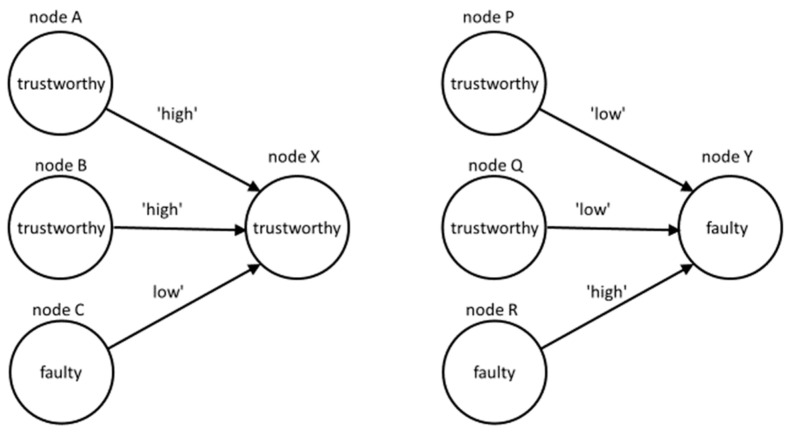
Example of two types of node configurations in the UOG.

Figure 2 shows two fragments of the UOG, where p=2 and q=1. On the left side, nodes A, B, and C provide opinions about the trustworthy node X. On the right side, nodes P, Q, and R provide opinions about the faulty node Y. In both cases, all the nodes also provide opinions to other nodes and are assessed by other nodes, but those opinions are not shown.

It is characteristic to this UOG that only those two configurations are possible: either the node is trustworthy, or it is faulty, and its role fully determines its behavior. This observation, together with some simplifications already introduced into the UOG, allows for simplifications in the description of the behavior of the reputation of its nodes.

- There are only two types of nodes, and they always receive the same amount of opinions from nodes of known types at regular intervals. Therefore, if the reputation of nodes at step *i* is known, it is possible to calculate the reputation of nodes at step *i* + 1 by calculating the reputation of one representative node from each type;
- Opinions depend solely on the type of nodes that provide and receive those opinions, so that trust expressed by those opinions can be determined in advance and is identical for every step;
- As the situation does not change between iterations, long-term decay can be incorporated into levels of trust that nodes report in opinions.

As a result of those simplifications, the original Equation (1), as applied to the UOG, becomes a set of two equations: one applicable for the iterative assessment of the reputation of trustworthy nodes and another for faulty ones.

(2) $$\left\{\begin{array}{c} x_{i+1}=\frac{px_{i}t+qy_{i}f}{px_{i}+qy_{i}}\\ y_{i+1}=\frac{px_{i}f+qy_{i}t}{px_{i}+qy_{i}} \end{array}\right.$$

where

- $x_{i}$ reputation of a representative “trustworthy” node at the *i*-th iteration, which is also the reputation of every trustworthy node in the graph
- $y_{i}$ reputation of a representative faulty node at the *i*-th iteration, which is also the reputation of every faulty node in the graph
- p the number of trustworthy nodes that provide opinions about the node
- q the number of faulty nodes that provide opinions about the node
- t the level of trust that is reported either by the trustworthy node about another trustworthy node or by the faulty node about another faulty node
- f the level of trust that is reported either by the trustworthy node about the faulty node or by the faulty node about the trustworthy one.

Note that there are additional limitations on the values that variables can assume that come from the coding convention used throughout the literature [29], as well as from the application area. Therefore:

- p≥0;q≥0;p+q>0. As the equation describes the graph, there is the requirement that this graph contains at least one node. For empty graphs or for graphs without any edge, the convergence is not assured, yet those graphs hardly represent any useful IoT network;
- 0≤t≤1;0≤f≤1;t>f. There is a choice of coding conventions for expressing the level of trust. The one chosen here assumes that the level of trust is represented by the real number between 0 and 1 and that “trustworthy” is coded as the value higher than “faulty”. For other conventions, convergence has not been studied;
- Eventually, $0\leq x_{i}\leq 1;0\leq y_{i}\leq 1$. The outcome of the calculation should be the reputation of the node, and the coding of such reputation should follow the same limitations as the coding of trust.

## 5. The Analysis of Convergence in the UOG

**Figure 3:**
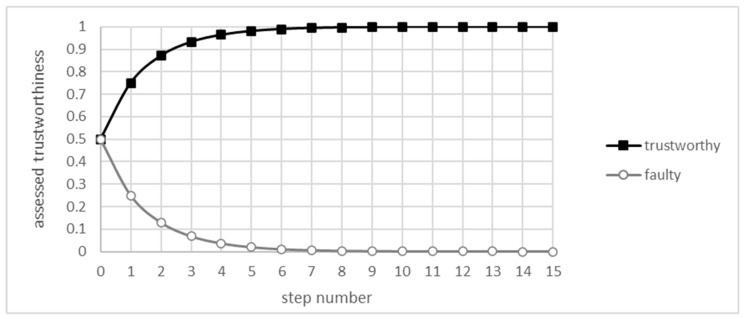
First steps of the algorithm for the UOG.

The simplification provided by the UOG allowed for the calculation of the convergence of the EWMA algorithm. Figure 3. shows subsequent values of $x_{i}$ and $y_{i}$ for the first 15 iterations of the algorithm defined by Equation (2), where parameters are p=3, q=1, t=1, and f=0, with the decay constant λ set to 0.7. Both functions seem to converge on values that do not seem to be far off from the values of *t* and *f*, respectively.

**Figure 4:**
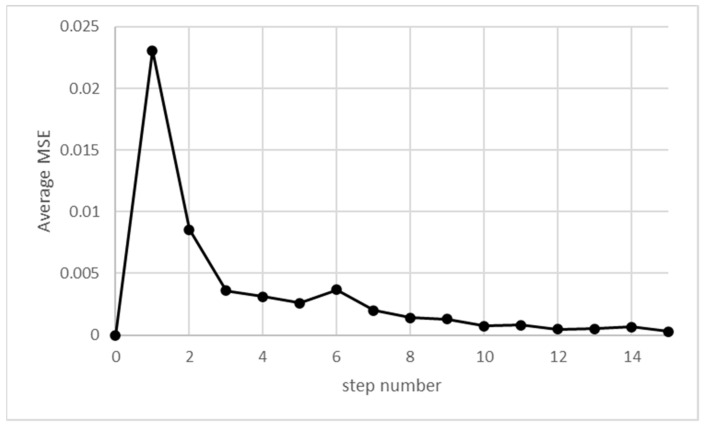
MSE between simulated and analytical results as a function of simulation steps.

The overall shape is very similar to the one presented in simulations provided in the work of [15], except for the lack of fluctuations, as there is no randomness in this analysis. Comparing with simulations using the same set of parameters, the difference between simulated and calculated results rapidly diminishes. The loss function, e.g., the MSE (Mean Squared Error), rapidly decreases, as shown in Figure 4.

While the comparison of analytical data with simulation can provide only limited assurance, this comparison demonstrates that, indeed, the UOG can serve as a suitable model of what occurs in the random graph when the EWMA algorithm is used.

Note that while there is a visible convergence in the assessment of both trustworthy and faulty nodes, they do not converge at *t* and *f*, respectively. Actual values (x*, y*) that the analytical formula derived from the UOG converges to can be determined by solving the set of Equation (3).

(3) $$\left\{\begin{array}{c} x^{*}=\frac{px^{*}t+qy^{*}f}{px^{*}+qy^{*}}\\ y^{*}=\frac{px^{*}f+qy^{*}t}{px^{*}+qy^{*}} \end{array}\right.$$

where

- x* the convergence value for the reputation of the representative trustworthy (“good”) node, which is also the convergence value of every trustworthy node in the UOG;
- y* the convergence value for the reputation of the representative faulty (bad) node, which is also the convergence value of every faulty node in the UOG.

## 6. The Proof of Convergence

This section demonstrates that for a non-trivial, realistic set of parameters, the set of functions (2) is convergent to its fixed point. The value of this fixed point is determined by the function’s parameters, but it always lies within their useful range. For reference, those ranges were defined as p≥0;q≥0;p+q>0, 0≤t≤1;0≤f≤1, and $0\leq x_{i}\leq 1;0\leq y_{i}\leq 1$. They refer to the real-life limitations already described earlier in this paper.

Referring back to the model, “trivial” cases are those that effectively preclude the use of the algorithm in the first place because of the lack of differentiation. These are the cases where either there is no differentiation between nodes (all are of the same type) or where there is no differentiation between opinions about nodes (i.e., both *t* and *f* are zero). Otherwise, the ability to converge for all non-trivial cases depends only on the choice of *t* and *f*, both being a coding convention only. In practice, *t* is assumed to be greater than *f*; e.g., throughout most of this paper, it was usually assumed that t=1 and f=0.

The proof defines the interval

$$M=\left[\max\left\{0,\;t+f-1\right\}\ldots \min\left\{1,\;t+f\right\}\right].$$

where

- *M* the interval within which the function converges so that all subsequent values of *x_i_* and *y_i_* belong to this interval;
- max{}, min{} maximum and minimum functions over the set of parameters.

It demonstrates that for f≠0 and t≠0, the sequence ${\left(x_{i}\right)}_{i=0\ldots \infty }$ starting from any $x_{0}\in M$ is convergent to a single fixed point in M. For t=0 (and f≠0), the sequence ${\left(x_{i}\right)}_{i=0\ldots \infty }$ oscillates around a single fixed point and is convergent only when it is constant and starts exactly from that fixed point. For f=0 (and t≠0) and p≠q the sequence ${\left(x_{i}\right)}_{i=0\ldots \infty }$ is always convergent to one end of the interval M (attractive fixed point), while the other end is also a fixed point, but only reachable by the constant sequence starting exactly from this point (repulsive fixed point). For f=0 (and t≠0) and p=q every point of the interval M is a fixed point and every sequence ${\left(x_{i}\right)}_{i=0\ldots \infty }$ starting from any $x_{0}\in M$ is always constant and converges to this point. For all cases, if the starting point $x_{0}$ lies outside the interval M (but is greater than zero), the first iteration, $x_{1}$, will fall into the interval M. Identical conclusions also apply to the sequence ${\left(y_{i}\right)}_{i=0\ldots \infty }$.

The proof starts with the reformulation of the problem, moving it from analyzing a set of two iterative functions into one iterative function (Section 6.1). Section 6.2, after dealing with trivial cases and potential truncation, demonstrates that the function has a support over the range M. The monotonicity of the function is then demonstrated, together with its two fixed points. It is demonstrated that only one of them lies within *M* and that it is the point to which the function converges (for f≠0 and t≠0). The remaining part of the proof (Section 6.3 onwards) demonstrates the convergence by demonstrating that values returned by the function from its subsequent iterations form a Cauchy sequence in a complete metric space M.

### 6.1. The Formulation of the Problem

The starting point of the proof is Equation (2), which contains a pair of iterative functions $x_{i}$ and $y_{i}$ representing the reputation of a trustworthy (high-quality) and a faulty (low-quality) node, respectively. Those functions have four parameters: *p, q, t*, and *f*, described earlier in this paper. The parameters meet the following conditions: p≥0, q≥0, and p+q>0 (there is at least one node), and $t,\;f\in \left[0,\;1\right]$. Additionally, we assume that $x_{0}\geq 0,\;y_{0}\geq 0$.

This pair of functions is a special case of a pair of linear fractional transformations that belong to the family of homographic functions. The behavior of this class of functions is known to strongly depend on the actual values of parameters. Therefore, the proof will concentrate on the behavior of those functions with respect to the values of their parameters.

**Conversion**. Note that $x_{i+1}+y_{i+1}=t+f$ for i=0…∞. This means that it is enough to analyze the one-dimensional sequence ${\left(x_{i}\right)}_{i=0\ldots \infty }$ that starts at *x_0_*, defined as

(4) $$\left\{\begin{array}{c} x_{0}\geq 0,\\ x_{i+1}=\frac{px_{i}t+q\left(t+f-x_{i}\right)f}{px_{i}+q\left(t+f-x_{i}\right)}=\frac{\left(pt-qf\right)x_{i}+q\left(t+f\right)f}{\left(p-q\right)x_{i}+q\left(t+f\right)}. \end{array}\right.$$

**Truncation**. Note, that $x_{i}+y_{i}=t+f$ for i>0. However, this does not always apply to $\left(x_{0},y_{0}\right)$. This irregularity is limited only to $\left(x_{0},y_{0}\right)$ as $\left(x_{1},y_{1}\right)$ always satisfy the equation. It has been already mentioned, as an observation, earlier in this paper. It is, therefore, enough to disregard the first element of a sequence without losing the generality.

**Trivial cases**. Let us consider the following trivial cases (for i=0…∞, for any $x_{0}\geq 0,\;y_{0}\geq 0$):

$$p=0\;\left(\mathrm{but}\;q\neq 0\right)\;\to \;x_{i+1}=f,\;y_{i+1}=t,$$

$$q=0\;\left(\mathrm{but}\;p\neq 0\right)\;\to \;x_{i+1}=t,\;y_{i+1}=f,$$

$$t=0\;\&\;f=0\;\left(i.e.\;t+f=0\right)\;\to \;x_{i+1}=0,\;y_{i+1}=0.$$

In all these cases, the sequences ${\left(x_{i}\right)}_{i=0\ldots \infty }$ and ${\left(y_{i}\right)}_{i=0\ldots \infty }$ are constant from i=1 onwards and converge to $x_{1}$ and $y_{1}$, respectively. Therefore, in further considerations, these cases can be excluded.

**Support over the range**. It is possible now to determine that the sequence ${\left(x_{i}\right)}_{i=0\ldots \infty }$ operates over a certain range in ℜ. It has been already assumed that $x_{i},y_{i}\geq 0;\;i=0\ldots \infty$ and p,q>0, and $t,f\in \left[0,\;1\right]$, but they cannot be zero at the same time (i.e., t+f>0). These conditions lead to the following inequalities: $0\leq px_{i}t+qy_{i}f\leq px_{i}+qy_{i}$ & $0\leq px_{i}f+qy_{i}t\leq px_{i}+qy_{i}$.

Expressions in both inequalities are nominators and denominators of (2), respectively. This means that $0\leq x_{i+1}\leq 1$ & $0\leq y_{i+1}\leq 1;\;i=0\ldots \infty$.

By replacing $y_{i+1}$ in the second inequality with the expression of $x_{i+1}$, an additional condition can be obtained $0\leq y_{i+1}=t+f-x_{i+1}\leq 1\;\to \;t+f-1\leq x_{i+1}\leq t+f;\;i=0\ldots \infty$.

This leads to the following bracketing condition that must be satisfied by every $x_{i}$ for i=1…∞:

$$m_{-}=\max\left\{0,t+f-1\right\}\leq x_{i}\leq \min\left\{1,t+f\right\}=m_{+};\;i=1\ldots \infty$$

where

- *m_−_* lower bracket (i.e., the minimum bracketing value) for any $x_{i}$;
- *m_+_* upper bracket (i.e., the maximum bracketing value) for any $x_{i}$.

With the truncation in mind, it is convenient to assume that $m_{-}\leq x_{0}\leq m_{+}$ as well. To simplify further the analysis, this paper will use the symbol M to denote the interval $\left[m_{-},\;m_{+}\right]$.

### 6.2. The Introduction of the Function

Note that for p≠q the equation that defines the sequence ${\left(x_{i}\right)}_{i=0\ldots \infty }$ determines the function 𝑔 defined as follows. This is the function that captures the iterative calculation of *x_i_* and *y_i_* from their previous values, but without cross-referencing, so that *x_i+1_* depends only on *x_i_* while *y_i+1_* depends only on *y_i_*

$$g:ℜ\backslash \left\{\frac{-q\left(t+f\right)}{p-q}\right\}\to ℜ,\;g\left(x\right)=\frac{\left(pt-qf\right)x+q\left(t+f\right)f}{\left(p-q\right)x+q\left(t+f\right)}.$$

For p=q the function 𝑔 becomes linear (as it was assumed that t+f>0):

$$g:ℜ\to ℜ,\;g\left(x\right)=\frac{t-f}{t+f}x+f.$$

Now the series ${\left(x_{i}\right)}_{i=0\ldots \infty }$ can be expressed as $x_{i+1}=g\left(x_{i}\right),\;y_{i+1}=t+f-g\left(t+f-y_{i}\right)$.

#### 6.2.1. Support over *M*

It was already established that it is enough to consider the interval $m_{-}\leq x\leq m_{+}$ and a function with a support restricted to this interval. If p>q, then the zero of the 𝑔’s denominator $\frac{-q\left(t+f\right)}{p-q}\leq 0\leq m_{-}$. If p<q, then the zero of the 𝑔’s denominator $\frac{-q\left(t+f\right)}{p-q}\geq t+f\geq m_{+}$ because $-q\left(t+f\right)\leq \left(p-q\right)\left(t+f\right)$. For p=q there is no risk of zero in the denominator (if only t+f≠0). Therefore, zero of the 𝑔’s denominator is never inside the interval $M=\left[m_{-},\;m_{+}\right]$ and we can consider the function 𝑔 in this interval without further considering its denominator equal to zero. Therefore, $g:M\to M,\;g\left(x\right)=\frac{\left(pt-qf\right)x+q\left(t+f\right)f}{\left(p-q\right)x+q\left(t+f\right)}$.

#### 6.2.2. Monotonicity

For *p* ≠ *q*, the function 𝑔 is a homographic transformation continuous in the interval *M*. Therefore, the function 𝑔 and its first and second derivatives 𝑔′ and 𝑔″ can be defined as follows:

$$g^{\prime }:M\to ℜ,\;g^{\prime }\left(x\right)=\frac{pq\left(t^{2}-f^{2}\right)}{{\left(\left(p-q\right)x+q\left(t+f\right)\right)}^{2}},$$

$$g^{\prime \prime }:M\to ℜ,\;g^{\prime \prime }\left(x\right)=\frac{-2pq\left(p-q\right)\left(t^{2}-f^{2}\right)}{{\left(\left(p-q\right)x+q\left(t+f\right)\right)}^{3}}$$

are strictly monotone in the domain M. Hence, extreme values (maximum and minimum) of both derivatives are reached at the ends of the interval M: for $m_{-}$ and $m_{+}$.

Further, if p=q then 𝑔 is linear, and its first derivative is constant, so its second derivative is equal to zero

$$g^{\prime }\left(x\right)=\frac{t-f}{t+f},\;g^{\prime \prime }\left(x\right)=0.$$

#### 6.2.3. Fixed Points

Fixed points of the equation $x=g\left(x\right)$ that are in M are as follows. For p=q and f≠0 there is one fixed point $x^{*}=\frac{t+f}{2}\in M$ as a solution of a linear equation $x=\frac{t-f}{t+f}x+f$. For p=q and f=0, t≠0 the function 𝑔 is an identity function $g\left(x\right)\equiv x$, and the whole interval M becomes a set of fixed points. For p≠q there can be no more than two fixed points on the real numbers axis because the function 𝑔 is a homographic transformation. Solving the equation $x=g\left(x\right)$, i.e.,

$$x=\frac{\left(pt-qf\right)x+q\left(t+f\right)f}{\left(p-q\right)x+q\left(t+f\right)}$$

leads to the formula:

$$x_{\pm }^{*}=\frac{-2qf+\left(p-q\right)t\pm \sqrt{4pqf^{2}+{\left(p-q\right)}^{2}t^{2}}}{2\left(p-q\right)}.$$

It remains to be demonstrated that for a minus sign before the square root, the fixed point $x_{-}^{*}\notin M$, assuming f>0; for a plus sign $x_{+}^{*}\in M$.

Note that throughout the rest of the text, fixed points will be denoted as follows:

- $x_{+}^{*}$ the fixed point calculated by using the plus sign in the above equation,
- $x_{-}^{*}$ the fixed point calculated by using the minus sign in the above equation,
- $x_{\pm }^{*}$ the pair of $x_{+}^{*}$ and $x_{-}^{*}$.

#### 6.2.4. $x_{-}^{*},x_{+}^{*}\in M$ for *f* = 0

For f=0 the fixed points of function 𝑔 are equal to:

$$x_{\pm }^{*}=\frac{\left(p-q\right)t\pm \sqrt{{\left(p-q\right)}^{2}t^{2}}}{2\left(p-q\right)}=\frac{\left(p-q\right)\pm \left|p-q\right|}{2\left(p-q\right)}t$$

and they are the ends of the interval *M*, which is equal to [0, *t*] when *f* = 0: $x_{-}^{*}=0=m_{-},\;x_{+}^{*}=t=m_{+}$ for *p* > *q* or $x_{-}^{*}=t=m_{+},\;x_{+}^{*}=0=m_{-}$ for *p* < *q*.

#### 6.2.5. $x_{-}^{*}\notin M$ for *f* > 0

To demonstrate that $x_{-}^{*}$ ∉ *M* for *f* > 0, let us assume the opposite: $x_{-}^{*}$ ∉ *M*. Then $x_{-}^{*}\geq m_{-}\geq 0$ and $x_{-}^{*}\leq m_{+}\leq t+f\;\to \;y_{-}^{*}=t+f-x_{-}^{*}\geq 0$. Consider the product: $x_{-}^{*}\cdot y_{-}^{*}=x_{-}^{*}\cdot \left(t+f-x_{-}^{*}\right)=-\frac{f}{2{\left(p-q\right)}^{2}}\cdot \left(4pqf-{\left(p-q\right)}^{2}t+\left(p+q\right)\sqrt{4pqf^{2}+{\left(p-q\right)}^{2}t^{2}}\right)$.

We introduce *ξ* as $\xi =4pqf-{\left(p-q\right)}^{2}t+\left(p+q\right)\sqrt{4pqf^{2}+{\left(p-q\right)}^{2}t^{2}}$. We estimate the expression described by a square root $\sqrt{4pqf^{2}+{\left(p-q\right)}^{2}t^{2}}\geq \left|p-q\right|t$. Hence for *p* > *q*: $\xi \geq 4pqf-{\left(p-q\right)}^{2}t+\left(p+q\right)\left(p-q\right)t=4pqf+2qt\left(p-q\right)>0$ and for *p* < *q* (*p* = *q* does not apply to this case): $\xi \geq 4pqf-{\left(q-p\right)}^{2}t+\left(p+q\right)\left(q-p\right)t=4pqf+2pt\left(q-p\right)>0$.

So the product of $x_{-}^{*}\cdot y_{-}^{*}=-\frac{f}{2{\left(p-q\right)}^{2}}\cdot \xi$, *ξ* < 0, if only *f* > 0, but a product of two non-negative numbers cannot be negative. It means that for *f* > 0, there must be $x_{-}^{*}$ ∉ *M*.

#### 6.2.6. $x_{+}^{*}\in M$ for *f* > 0

Let us show that $x_{-}^{*}$ ∉ *M* for *f* > 0 (for *f* = 0 we have just shown it). It means that we should prove: $m_{-}=\max\left\{0,t+f-1\right\}\leq x_{+}^{*}\leq \min\left\{1,t+f\right\}=m_{+}$.

We will prove in turn that (1) $x_{+}^{*}\geq 0$, and (2) $x_{+}^{*}\geq t+f-1$, and (3) $x_{+}^{*}\leq t+f$ and finally (4) $x_{+}^{*}\leq 1$. We will prove each of these inequalities for two cases: *p* > *q* and *p* < *q* (bearing in mind that the case *p* = *q* has been considered separately, and now it can be omitted).

**Lemma** **1.** $x_{+}^{*}\geq 0$*. For p > q, we obtain estimates (notice that then*$p>\sqrt{pq}>q$):

$$x_{+}^{*}=\frac{-2qf+\left(p-q\right)t+\sqrt{4pqf^{2}+{\left(p-q\right)}^{2}t^{2}}}{2\left(p-q\right)}\geq \frac{\left(p-q\right)t+2\left(\sqrt{pq}-q\right)f}{2\left(p-q\right)}>\frac{t}{2}\geq 0$$

*For p < q, we obtain estimates (notice that then*$p<\sqrt{pq}<q$):

$$x_{+}^{*}=\frac{-2qf+\left(p-q\right)t+\sqrt{4pqf^{2}+{\left(p-q\right)}^{2}t^{2}}}{2\left(p-q\right)}\geq \frac{\left(q-\sqrt{pq}\right)f}{q-p}\geq 0.$$

*Therefore*$x_{+}^{*}\geq 0$.

**Lemma** **2.** $x_{+}^{*}\geq t+f-1$.

This lemma will be considered in two steps: for *p* > *q*; *t* + *f* > 1; and for *p* < *q*; *t* + *f* > 1. Let us notice first that the proof is necessary only for *t* + *f* > 1; as for the opposite, Lemma 1 applies. For the case of *p* > *q* and *t* + *f* > 1, it is possible to write (because all subtractions provide non-negative results):

$$4\left(p-q\right)\left(q\left(1-t\right)+p\left(1-f\right)\left(t+f-1\right)\right)\geq 0,$$

so that

$$4pqf^{2}+{\left(p-q\right)}^{2}t^{2}\geq {\left(\left(p-q\right)\left(t-2\right)+2pf\right)}^{2}.$$

Because $4pqf^{2}+{\left(p-q\right)}^{2}t^{2}\geq 0$, square root can be applied to both sides of the last inequality:

$$\sqrt{4pqf^{2}+{\left(p-q\right)}^{2}t^{2}}\geq \left|\left(p-q\right)\left(t-2\right)+2pf\right|\geq \left(p-q\right)\left(t-2\right)+2pf,$$

so that

$$x_{+}^{*}=\frac{-2qf+\left(p-q\right)t+\sqrt{4pqf^{2}+{\left(p-q\right)}^{2}t^{2}}}{2\left(p-q\right)}\geq t+f-1,$$

therefore,

$$x_{+}^{*}\geq t+f-1.$$

For the case of *p* < *q* (and *t* + *f* > 1) it is possible to write (because only *p* − *q* < 0 and all other subtractions provide non-negative results):

$$4\left(p-q\right)\left(q\left(1-t\right)+p\left(1-f\right)\left(t+f-1\right)\right)\leq 0,$$

so that

$$4pqf^{2}+{\left(p-q\right)}^{2}t^{2}\leq {\left(\left(p-q\right)\left(t-2\right)+2pf\right)}^{2}.$$

Because $4pqf^{2}+{\left(p-q\right)}^{2}t^{2}\geq 0$ and (*p* − *q*)(*t* – 2) + 2*pf* = (*q* − *p*)(2 − *t*) + 2*pf* ≥ 0 square root can be applied to both sides of the last inequality

$$\sqrt{4pqf^{2}+{\left(p-q\right)}^{2}t^{2}}\leq \left(p-q\right)\left(t-2\right)+2pf,$$

so that

$$x_{+}^{*}=\frac{-2qf+\left(p-q\right)t+\sqrt{4pqf^{2}+{\left(p-q\right)}^{2}t^{2}}}{2\left(p-q\right)}\geq t+f-1,$$

hence,

$$x_{+}^{*}\geq t+f-1.$$

Lemma 1 and Lemma 2 demonstrate that $x_{+}^{*}\geq m_{-}=\max\left\{0,t+f-1\right\}$. Now it remains to show that $x_{+}^{*}\leq m_{+}$. It is performed in two steps: Lemma 3 and Lemma 4.

**Lemma** **3.** $x_{+}^{*}\leq t+f$.

For *p* > *q*, $p-q=\left(\sqrt{p}+\sqrt{q}\right)\left(\sqrt{p}-\sqrt{q}\right)>2\sqrt{q}\left(\sqrt{p}-\sqrt{q}\right)=2\left(\sqrt{pq}-q\right)$, so that:

$$x_{+}^{*}=\frac{-2qf+\left(p-q\right)t+\sqrt{4pqf^{2}+{\left(p-q\right)}^{2}t^{2}}}{2\left(p-q\right)}\;\leq \frac{2\left(p-q\right)t+2\left(\sqrt{pq}-q\right)f}{2\left(p-q\right)}\;<\frac{2\left(p-q\right)t+\left(p-q\right)f}{2\left(p-q\right)}=t+\frac{f}{2}<t+f.$$

For *p* < *q*, in a similar way, $q-p=\left(\sqrt{q}+\sqrt{p}\right)\left(\sqrt{q}-\sqrt{p}\right)>\sqrt{q}\left(\sqrt{q}-\sqrt{p}\right)=q-\sqrt{pq}$, so that:

$$x_{+}^{*}=\frac{-2qf+\left(p-q\right)t+\sqrt{4pqf^{2}+{\left(p-q\right)}^{2}t^{2}}}{2\left(p-q\right)}\;\leq \frac{\left(q-p\right)t+2\left(q-\sqrt{pq}\right)f}{2\left(q-p\right)}\;<\frac{\left(q-p\right)t+2\left(q-p\right)f}{2\left(q-p\right)}=\frac{t}{2}+f\leq t+f.$$

**Lemma** **4.** $x_{+}^{*}\leq 1$.

It has been shown that $x_{+}^{*}\leq t+f$, so to show that $x_{+}^{*}\leq 1$ it is enough to consider *t* + *f* > 1. Note that for *p* > *q* and *t* + *f* > 1, we can write (because all subtractions provide non-negative results):

$$4{\left(p-q\right)}^{2}\left(1-t\right)+4\left(p-q\right)qf\left(2-t-f\right)\geq 0,\;{\left(\left(p-q\right)\left(2-t\right)+2qf\right)}^{2}\geq 4pqf^{2}+{\left(p-q\right)}^{2}t^{2},\;\frac{-2qf+\left(p-q\right)t+\sqrt{4pqf^{2}+{\left(p-q\right)}^{2}t^{2}}}{2\left(p-q\right)}\leq 1,\;x_{+}^{*}\leq 1.$$

For *p* < *q* and *t* + *f* > 1, we can write (because only *p* – *q* < 0 and all other subtractions provide non-negative results):

$$4\left(p-q\right)\left(p\left(1-t\right)+q\left(1-f\right)\left(t+f-1\right)\right)\leq 0,\;{\left(\left(p-q\right)\left(2-t\right)+2qf\right)}^{2}\leq 4pqf^{2}+{\left(p-q\right)}^{2}t^{2},\;\frac{-2qf+\left(p-q\right)t+\sqrt{4pqf^{2}+{\left(p-q\right)}^{2}t^{2}}}{2\left(p-q\right)}\leq 1,\;x_{+}^{*}\leq 1.$$

Lemmas 1 to 4 demonstrate that $m_{-}=\max\left\{0,t+f-1\right\}\leq x_{+}^{*}\leq \min\left\{1,t+f\right\}=m_{+}$ so that $x_{+}^{*}\in M$. Moreover, for *f* > 0, $x_{+}^{*}$ is the only fixed point of the function 𝑔 in the interval M.

### 6.3. Convergence

Now it is possible to prove that for *t* ≠ 0 and *f* ≠ 0, any sequence (*x_i_*)_*i*=0…∞_ starting with $x_{0}$ ≥ 0 is convergent to a single fixed point $x_{+}^{*}$ ∈ *M*, denoted below as x*. Of course, this also implies the convergence of the sequence (*y_i_*)_*i*=0…∞_ starting with $y_{0}$ ≥ 0 to y* = *t* + *f* − x* ∈ *M*.

One has to remember that we have assumed that *x_i_* ≥ 0 (where *i* = 0…∞) and *p*, *q* > 0, and *t*, *f* ∈ [0, 1], but they cannot be zero at the same time (i.e., *t* + *f* > 0). Because we demonstrated that $x_{1}$ ∈ *M* for any $x_{0}$ ≥ 0, we can limit our considerations to $x_{0}$ ∈ *M* (in a way, we can treat $x_{1}$ as $x_{0}$).

The proof of convergence applies different methods depending on the ranges of values of parameters. It starts from some simple cases and then demonstrates convergence either by determining that the function is a concave function or by demonstrating that subsequent iterations form a Cauchy sequence.

#### 6.3.1. The Case of t=0 and f≠0

This case uses the coding that is not typical, with “good” being reported as zero and “bad” as non-zero (effectively as greater than zero). This kind of coding leads to the lack of convergence and should be excluded.

It will be now demonstrated that for *t* = 0 and *f* ≠ 0, subsequent values of $x_{i}$ alternate between two values so that the function does not converge. It will allow excluding this case from further considerations. Note that this case counters the assumption about the rationality of the coding: trustworthiness is here encoded as zero, and fault as a value greater than zero. We have:

$$g\left(x\right)=\frac{qf\left(f-x\right)}{\left(p-q\right)x+qf}$$

Let us calculate $x_{i+2}=g\left(x_{i+1}\right)=g\left(g\left(x_{i}\right)\right)$:

$$x_{i+2}=\frac{qf\left(f-x_{i+1}\right)}{\left(p-q\right)x_{i+1}+qf}=\frac{qf\left(f-\frac{qf\left(f-x_{i}\right)}{\left(p-q\right)x_{i}+qf}\right)}{\left(p-q\right)\frac{qf\left(f-x_{i}\right)}{\left(p-q\right)x_{i}+qf}+qf}\;=\frac{\left(p-q\right)qf^{2}x_{i}+q^{2}f^{2}x_{i}}{\left(p-q\right)qf^{2}+q^{2}f^{2}}=x_{i}.$$

So for *t* = 0, the iteration sequence takes the form of $x_{0},\;x_{1},\;x_{0},\;x_{1},\;x_{0},\;x_{1}$... and is not convergent unless it is constant, i.e., $x_{i}=x^{*}$ for *i* = 0…∞. All the subsequent cases assume that t≠0.

#### 6.3.2. The Case of f=0 and t≠0 and p≠q

This case refers to the situation where the coding scheme has been chosen in a rational way and where the number of “good” nodes is not equal to the number of “bad” ones. Therefore, it is the case that may be considered typical. Note that it is not relevant for the convergence whether there are more “bad” or “good” nodes, even though it may lead to unexpected results.

In order to demonstrate the convergence of 𝑔(*x*) for *f* = 0 and *t* ≠ 0 and *p* ≠ *q*, the sequence ${\left(\alpha_{i}\right)}_{i=0\ldots \infty }$ is introduced. For *f* = 0 and the sequence ${\left(x_{i}\right)}_{i=0\ldots \infty }$, where $x_{i+1}=g\left(x_{i}\right)$, it is possible to describe its every element belonging to the interval *M* = [0, *t*] as $x_{i}$ = $\alpha_{i}$ · *t*, where $\alpha_{i}$ ∈ [0, 1].

$$x_{i+1}=\frac{px_{i}t}{\left(p-q\right)x_{i}+qt}=\frac{p\alpha_{i}t^{2}}{\left(p-q\right)\alpha_{i}t+qt}=\frac{p\alpha_{i}t}{\left(p-q\right)\alpha_{i}+q}=\alpha_{i+1}t$$

The corresponding sequence ${\left(\alpha_{i}\right)}_{i=0\ldots \infty }$ is as follows:

$$\alpha_{i+1}=\frac{p\alpha_{i}}{\left(p-q\right)\alpha_{i}+q}=\frac{p\alpha_{i}}{p\alpha_{i}+q\left(1-\alpha_{i}\right)}.$$

To prove the convergence of the sequence ${\left(x_{i}\right)}_{i=0\ldots \infty }$ for any $x_{0}=\alpha_{0}\cdot t$ ∈ *M* = [0, *t*] it is enough to show the convergence of the sequence ${\left(\alpha_{i}\right)}_{i=0\ldots \infty }$ for any $\alpha_{0}$ ∈ [0, 1].

The sequence ${\left(\alpha_{i}\right)}_{i=0\ldots \infty }$ can also be defined without resorting to recursion:

$$\alpha_{i}=\frac{p^{i}\alpha_{0}}{\left(p^{i}-q^{i}\right)\alpha_{0}+q^{i}}=\frac{p^{i}\alpha_{0}}{p^{i}\alpha_{0}+q^{i}\left(1-\alpha_{0}\right)}=\frac{\alpha_{0}}{\alpha_{0}+{\left(\frac{q}{p}\right)}^{i}\left(1-\alpha_{0}\right)}$$

which is easy to prove by mathematical induction.

If $\alpha_{0}$ = 0 ($x_{0}$ = 0) then $\alpha_{i}$ = 0 for *i* = 0…∞, which is easy to prove by mathematical induction.

If $\alpha_{0}$ = 0 ($x_{0}$ = 0) then $\alpha_{i}$ = 0 for *i* = 0…∞ and both sequences ($\alpha_{i}$)_*i*=0…∞_ and ($x_{i}$)_*i*=0…∞_ are constant and converge to 0. If $\alpha_{0}$ = 1 ($x_{0}$ = *t*) then $\alpha_{i}$ = 1 for *i* = 0…∞ and the sequences ($\alpha_{i}$)_*i*=0…∞_ and ($x_{i}$)_*i*=0…∞_ are constant and convergent to 1 and *t*, respectively.

For $\alpha_{i}$ ∈ (0, 1), two cases should be considered (note the assumption that *p* ≠ *q*):

$$p>q\;\to \;\underset{i\to \infty }{\lim}{\left(\frac{q}{p}\right)}^{i}=0\;\to \;\underset{i\to \infty }{\lim}\alpha_{i}=\underset{i\to \infty }{\lim}\frac{\alpha_{0}}{\alpha_{0}+{\left(\frac{q}{p}\right)}^{i}\left(1-\alpha_{0}\right)}=1\;\to \;\underset{i\to \infty }{\lim}x_{i}=t,\;p<q\;\to \;\underset{i\to \infty }{\lim}{\left(\frac{q}{p}\right)}^{i}=\infty \;\to \;\underset{i\to \infty }{\lim}\alpha_{i}=\underset{i\to \infty }{\lim}\frac{\alpha_{0}}{\alpha_{0}+{\left(\frac{q}{p}\right)}^{i}\left(1-\alpha_{0}\right)}=0\;\to \;\underset{i\to \infty }{\lim}x_{i}=0.$$

Combining both cases together, we obtain that for *p* ≠ *q* and 0 < $x_{0}$ < *t*, the sequence (*x_i_*)_*i*=0…∞_ is always convergent to $x_{+}^{*}$.

It means that for *f* = 0 and *t* ≠ 0 and *p* ≠ *q* the sequence (*x_i_*)_*i*=0…∞_ is always convergent to $x_{+}^{*}$ if $x_{0}$ ∈ *M*\{$x_{-}^{*}$} (i.e., $x_{0}\neq x_{-}^{*}$) or to $x_{-}^{*}$ if $x_{0}=x_{-}^{*}$ (the constant sequence $x_{i}=x_{-}^{*}$).

#### 6.3.3. The Case of f=0 and t≠0 and p=q

This is the case of the rational coding scheme, but of the specific network where the number of “good” and “bad” nodes is equal. For reference, the actual simulation (not the UOG) shows that the function randomly “wobbles” around a certain fixed level.

For *f* = 0 and *t* ≠ 0 and *p* = *q*, the function 𝑔 is an identity function $g\left(x\right)\equiv x$, and a set of fixed points is the whole interval *M*. This means that any sequence $x_{i+1}=g\left(x_{i}\right)$ for *i* = 0…∞ is constant and convergent to $x_{x_{0}}^{*}=x_{0}$.

#### 6.3.4. The Case of f≠0 and t≠0 and p=q

This case considers the situation of the network with equal numbers of “good” and “bad” nodes, where the coding is rationally chosen. In a manner similar to the previous case, the actual simulation shows the function “wobbles” around a certain value. For the UOG, this value can be determined from the following analysis.

We have shown previously that for *f* ≠ 0 and *t* ≠ 0 and *p* = *q*, a fixed point is x* = $\frac{t+f}{2}$ ∈ *M*. However, we have not shown how the sequence (*x_i_*)_*i*=0…∞_ behaves for this case:

$$x_{i+1}=g\left(x_{i}\right)=\frac{t-f}{t+f}x_{i}+f.$$

This sequence can also be defined not recursively:

$$x_{i}={\left(\frac{t-f}{t+f}\right)}^{i}x_{0}+f\overset{i-1}{\underset{k=0}{\sum }}{\left(\frac{t-f}{t+f}\right)}^{k}={\left(\frac{t-f}{t+f}\right)}^{i}x_{0}+f\frac{1-{\left(\frac{t-f}{t+f}\right)}^{i-1}}{1-\frac{t-f}{t+f}}$$

which can be easily proved by mathematical induction.

This case of the current concern is *f* ≠ 0. We have already considered separately the case *t* = 0, so now we can assume that *t* ≠ 0. For *t*, *f* > 0 we have −*t* − *f* < *t* − *f* < *t* + *f* and $\left|\frac{t-f}{t+f}\right|<1$. This means that:

$$\underset{i\to \infty }{\lim}{\left(\frac{t-f}{t+f}\right)}^{i}=0.$$

Hence for the case *f* ≠ 0 and *t* ≠ 0 and *p* = *q*:

$$\underset{i\to \infty }{\lim}x_{i}=\underset{i\to \infty }{\lim}\left({\left(\frac{t-f}{t+f}\right)}^{i}x_{0}+f\frac{1-{\left(\frac{t-f}{t+f}\right)}^{i-1}}{1-\frac{t-f}{t+f}}\right)\;=f\frac{1}{1-\frac{t-f}{t+f}}=\frac{f\left(t+f\right)}{t+f-t+f}=\frac{t+f}{2}=x^{*}.$$

and both sequences ($\alpha_{i}$)_*i*=0…∞_ and ($x_{i}$)_*i*=0…∞_ are constant and converge to 0. If $\alpha_{0}$= 1 ($x_{0}$ = *t*) then $\alpha_{i}$ = 1 for *i* = 0…∞ and the sequences ($\alpha_{i}$)_*i*=0…∞_ and ($x_{i}$)_*i*=0…∞_ are constant and convergent to 1 and *t*, respectively.

For $\alpha_{i}$ ∈ (0, 1), two cases should be considered (note the assumption that *p* ≠ *q*):

$$p>q\;\to \;\underset{i\to \infty }{\lim}{\left(\frac{q}{p}\right)}^{i}=0\;\to \;\underset{i\to \infty }{\lim}\alpha_{i}=\underset{i\to \infty }{\lim}\frac{\alpha_{0}}{\alpha_{0}+{\left(\frac{q}{p}\right)}^{i}\left(1-\alpha_{0}\right)}=1\;\to \;\underset{i\to \infty }{\lim}x_{i}=t,\;p<q\;\to \;\underset{i\to \infty }{\lim}{\left(\frac{q}{p}\right)}^{i}=\infty \;\to \;\underset{i\to \infty }{\lim}\alpha_{i}=\underset{i\to \infty }{\lim}\frac{\alpha_{0}}{\alpha_{0}+{\left(\frac{q}{p}\right)}^{i}\left(1-\alpha_{0}\right)}=0\;\to \;\underset{i\to \infty }{\lim}x_{i}=0.$$

Combining both cases together, we obtain that for *p* ≠ *q* and 0 < $x_{0}$ < *t* the sequence ($x_{i}$)_*i*=0…∞_ is always convergent to $x_{+}^{*}$.

It means that for *f* = 0 and *t* ≠ 0 and *p* ≠ *q*, the sequence ($x_{i}$)_*i*=0…∞_ is always convergent to $x_{+}^{*}$ if $x_{0}$ ∈ *M*\{$x_{-}^{*}$}(i.e., $x_{0}\neq x_{-}^{*}$) or to $x_{-}^{*}$ if $x_{0}=x_{-}^{*}$ (the constant sequence $x_{i}=x_{-}^{*}$).

#### 6.3.5. The Case of f≠0 and t≠0 and p≠q

This case describes the network with an unequal number of “good” and “bad” nodes. In reference to Section 6.3.2, this case can also be called a typical one. Note that the fact of convergence does not depend on the choice of a coding scheme.

To prove the convergence of the sequence ($x_{i}$)_*i*=0…∞_ for *f* ≠ 0 and *t* ≠ 0 and *p* ≠ *q*, it is enough to show that the sequence ($x_{i}$)_*i*=0…∞_ is a Cauchy sequence (because *M* is a complete metric space).

First, we calculate (𝑔(*x*)):

$$g\left(g\left(x\right)\right)=\frac{\left(pt-qf\right)\frac{\left(pt-qf\right)x+q\left(t+f\right)f}{\left(p-q\right)x+q\left(t+f\right)}+q\left(t+f\right)f}{\left(p-q\right)\frac{\left(pt-qf\right)x+q\left(t+f\right)f}{\left(p-q\right)x+q\left(t+f\right)}+q\left(t+f\right)}\;=\frac{\left(pt\left(pt-qf\right)+qf\left(pf-qt\right)\right)x+q\left(p+q\right)\left(t+f\right)tf}{\left(p-q\right)\left(p+q\right)tx+q\left(pf+qt\right)\left(t+f\right)}.$$

Let us estimate a distance between $x_{i+2}$ and $x_{i+1}$ according to the distance between $x_{i+1}$ and $x_{i}$:

xi+1−xi=gxi−xi=pt−qfxi+qt+ff−p−qxi2−qt+fxip−qxi+qt+f=−p−qxi2+p−qt−2qfxi+qt+ffp−qxi+qt+f,xi+2−xi+1=ggxi−gxi=ptpt−qf+qfpf−qtxi+qp+qt+ftfp−qp+qtxi+qpf+qtt+f−pt−qfxi+qt+ffp−qxi+qt+f=pqt−ft+f·−p−qxi2+p−qt−2qfxi+qt+ffp−qp+qtxi+qpf+qtt+fa^?p−qxi+qt+f=pqt−ft+fp−qp+qtxi+qpf+qtt+fxi+1−xi.

Note that for $0\leq x_{i}\leq t+f$ the denominator is always positive:

$$\left(p-q\right)\left(p+q\right)tx_{i}+q\left(pf+qt\right)\left(t+f\right)>0.$$

It is obvious for *p* > *q*, while for *p* < *q*, it results from the inequality:

$$\left(pqf+q^{2}t\right)\left(t+f\right)>\left(q^{2}t-p^{2}t\right)\left(t+f\right)\geq \left(q^{2}t-p^{2}t\right)x_{i}$$

It is necessary at this point to break down the analysis into four more specific sub-cases, provided below as Section 6.3.6, Section 6.3.7, Section 6.3.8 and Section 6.3.9. It is because the way the convergence occurs depends on both the coding scheme as well as on the relative number of “good” and “bad” nodes.

#### 6.3.6. The Case of t<f and p>q

This is the case of the ill-chosen (yet possible) coding, where “good” is indicated by the lower value than “bad”. Further, the network has more “good” nodes than “bad” ones.

For *p* > *q* (and *x_i_* ≥ 0, *p* ≥ 0, *q* ≥ 0, *t* > 0):

$$\left|x_{i+2}-x_{i+1}\right|=\frac{pq\left|t-f\right|\left(t+f\right)}{\left(p-q\right)\left(p+q\right)tx_{i}+q\left(pf+qt\right)\left(t+f\right)}\left|x_{i+1}-x_{i}\right|\;\leq \frac{pq\left|t-f\right|\left(t+f\right)}{q\left(pf+qt\right)\left(t+f\right)}\left|x_{i+1}-x_{i}\right|\;=\frac{p\left|t-f\right|}{pf+qt}\left|x_{i+1}-x_{i}\right|.$$

If *t* < *f* (and *p* + *q* > 0, *t* > 0) then

$$k_{\pm }=\frac{p\left|t-f\right|}{pf+qt}=\frac{p\left(f-t\right)}{p\left(f-t\right)+\left(p+q\right)t}<1$$

because 0 < *p*(*f* − *t*) < *p*(*f* − *t*) + (*p* + *q*)*t*.

It means that for *p* > *q* and *t* < *f*, the sequence ($x_{i}$)_*i*=0…∞_ is a Cauchy sequence in the interval *M*, i.e., $\left|x_{i+2}-x_{i+1}\right|<k_{+}\left|x_{i+1}-x_{i}\right|$ for *i* = 0…∞ and some $k_{+}<1$, and it is convergent.

#### 6.3.7. The Case of t<f and p<q

This is the case of the ill-chosen (yet possible) coding, where “good” is indicated by the lower value than “bad”. Further, the network has more “bad” nodes than “good” ones. This situation may be hard for the outcome of the algorithm (as the excess of “bad” nodes may not allow for the correct identification of the quality of nodes), but it is important that the convergence still occurs.

For *p* < *q* (and $x_{i}$ ≤ *t* + *f*, *p* ≥ 0, *q* ≥ 0, *t* > 0) we have:

$$\left|x_{i+2}-x_{i+1}\right|=\frac{pq\left|t-f\right|\left(t+f\right)}{\left(p-q\right)\left(p+q\right)tx_{i}+q\left(pf+qt\right)\left(t+f\right)}\left|x_{i+1}-x_{i}\right|\;\leq \frac{pq\left|t-f\right|\left(t+f\right)}{\left(p-q\right)\left(p+q\right)t\left(t+f\right)+q\left(pf+qt\right)\left(t+f\right)}\left|x_{i+1}-x_{i}\right|\;=\frac{q\left|t-f\right|}{pt+qf}\left|x_{i+1}-x_{i}\right|.$$

If *t* < *f* (and *p* + *q* > 0, *t* > 0), then

$$k_{-}=\frac{q\left|t-f\right|}{pt+qf}=\frac{q\left(f-t\right)}{q\left(f-t\right)+\left(p+q\right)t}<1$$

because 0 < *q*(*f* − *t*) < *q*(*f* − *t*) + (*p* + *q*)*t*.

It means that for *p* < *q* and *t* < *f* the sequence ($x_{i}$)_*i*=0…∞_ is a Cauchy sequence in the interval *M*, i.e., $\left|x_{i+2}-x_{i+1}\right|<k_{-}\left|x_{i+1}-x_{i}\right|$ for *i* = 0…∞ and some $k_{-}<1$, and it is convergent.

#### 6.3.8. The Case of t>f and p>q

This is the case of the coding that may be considered a rational one, where “good” is indicated by a higher value than “bad”. Further, the network has more “good” nodes than “bad” ones. In a way, this is a typical and the desired situation.

We still have to consider case *t* > *f* and *p* ≠ *q*. Unfortunately, it is a bit more complicated and has to be split again into two cases, for *p* > *q* and *p* < *q*.

For *p* > *q* and *t* > *f*, the fixed point is $x^{*}>\frac{t+f}{2}$. From the obvious fact that for *t* > *f*:

$$4pqf^{2}+{\left(p-q\right)}^{2}t^{2}>{\left(p+q\right)}^{2}f^{2},$$

it is easy to show that

$$x^{*}=\frac{-2qf+\left(p-q\right)t+\sqrt{4pqf^{2}+{\left(p-q\right)}^{2}t^{2}}}{2\left(p-q\right)}>\frac{t+f}{2}.$$

For *p* > *q* and *t* > *f* the function $h\left(x\right)=g\left(x\right)-x$ is a concave function in the interval *M*. Indeed, its second-degree derivative is equal to 𝑔″(*x*) and negative in this interval:

$$h^{\prime \prime }\left(x\right)=g^{\prime \prime }\left(x\right)=\frac{-2pq\left(p-q\right)\left(t^{2}-f^{2}\right)}{{\left(\left(p-q\right)x+q\left(t+f\right)\right)}^{3}}<0.$$

This means that a minimum of this function is reached at one of the ends of a considered range. Therefore for the initial part of the interval *M*: $0\leq x_{i}\leq \frac{t+f}{2}$ we can estimate the distance $x_{i+1}-x_{i}$ from below by $\rho_{+}=\min\left\{g\left(0\right)-0,g\left(\frac{t+f}{2}\right)-\frac{t+f}{2}\right\}>0$, i.e.,

$$x_{i+1}-x_{i}\geq \rho_{+}=\min\left\{g\left(0\right)-0,g\left(\frac{t+f}{2}\right)-\frac{t+f}{2}\right\}>0.\;x_{i+1}-x_{i}=\frac{-\left(p-q\right)x_{i}^{2}+\left(\left(p-q\right)t-2qf\right)x_{i}+q\left(t+f\right)f}{\left(p-q\right)x_{i}+q\left(t+f\right)}\geq g\left(\frac{t+f}{2}\right)-\frac{t+f}{2}\;=\frac{\left(p-q\right)\left(t-f\right)}{2\left(p+q\right)}=\frac{p-q}{p+q}\cdot \frac{t-f}{2}>0.$$

$$x_{i+1}-x_{i}=\frac{-\left(p-q\right)x_{i}^{2}+\left(\left(p-q\right)t-2qf\right)x_{i}+q\left(t+f\right)f}{\left(p-q\right)x_{i}+q\left(t+f\right)}\geq g\left(0\right)-0=f>0$$

Hence,

$$x_{i+1}-x_{i}\geq \rho_{+}=\min\left\{f,\frac{p-q}{p+q}\cdot \frac{t-f}{2}\right\}>0,\;x_{i+1}>x_{i}.$$

So for $0\leq x_{i}\leq \frac{t+f}{2}$, the sequence ($x_{i}$)_*i*=0…∞_ is strictly increasing and while $x_{i}\leq \frac{t+f}{2}$:

$$x_{i+1}\geq x_{i}+\rho_{+}>x_{i}.$$

Thus if $0\leq x_{0}\leq \frac{t+f}{2}$ then $x_{1}\geq x_{0}+\rho_{+}\geq \rho_{+}>0$. Similarly, if $\rho_{+}\leq x_{1}\leq \frac{t+f}{2}$ then $x_{2}\geq x_{1}+\rho_{+}\geq 2\rho_{+}$. Generalizing, if $i\rho_{+}\leq x_{i}\leq \frac{t+f}{2}$ then $x_{i+1}\geq x_{i}+\rho_{+}\geq \left(i+1\right)\rho_{+}$. This means that if for *i* ≤ *n*, where $n=\lceil \frac{t+f}{2\rho_{+}}\rceil \geq \frac{t+f}{2\rho_{+}}$ (⌈⌉ denotes a ceiling function) all $x_{i}\leq \frac{t+f}{2}$, then $x_{n}\geq \left(n+1\right)\rho_{+}\geq \left(\frac{t+f}{2\rho_{+}}+1\right)\rho_{+}=\frac{t+f}{2}+\rho_{+}>\frac{t+f}{2}$. In other words, among the *n* + 1 first elements of the sequence ($x_{i}$)_*i*=0…∞_, there must appear an element greater than $\frac{t+f}{2}$. So some element of the sequence ($x_{i}$)_*i*=0…∞_ lies above $\frac{t+f}{2}$. Let us denote the first such element with the index *j*.

Now, assuming *t* > *f*, we can write for $x_{i}>\frac{t+f}{2}$:

$$\left|x_{i+2}-x_{i+1}\right|=\frac{pq\left(t-f\right)\left(t+f\right)}{\left(p-q\right)\left(p+q\right)tx_{i}+q\left(pf+qt\right)\left(t+f\right)}\left|x_{i+1}-x_{i}\right|\;<\frac{pq\left(t-f\right)\left(t+f\right)}{\left(p-q\right)\left(p+q\right)t\frac{1}{2}\left(t+f\right)+q\left(pf+qt\right)\left(t+f\right)}\left|x_{i+1}-x_{i}\right|\;=\frac{2pq\left(t-f\right)}{p^{2}t+q^{2}t+2pqf}\left|x_{i+1}-x_{i}\right|.$$

If *t* > *f* and $x_{i}>\left(t+f\right)/2$, then

$$k_{+}=\frac{2pq\left(t-f\right)}{p^{2}t+q^{2}t+2pqf}<1$$

because *p*²*t* + *q*²*t* + 2*pqf* > 2*pq*(*t* − *f*) > 0, since (*p* − *q*)²*t* > −4*pqf*.

It means that for *p* > *q* and *t* > *f*, the sequence ($x_{i}$)_*i*=0…∞_ is a Cauchy sequence in the interval *M*, i.e., $\left|x_{i+2}-x_{i+1}\right|<k_{+}\left|x_{i+1}-x_{i}\right|$ for *i* = *j*…∞ and some $k_{+}<1$, and it is convergent.

#### 6.3.9. The Case of t>f and p<q

This is the case of coding that can be considered rational, where “good” is indicated by a higher value than “bad”. However, the network has more “bad” nodes than “good” ones, so that the resulting value may not be as expected. Still, it is important that convergence occurs.

For *p* < *q* and *t* > *f*, the fixed point $x^{*}<\frac{t+f}{2}$. From the obvious fact that for *t* > *f*:

$$4pqf^{2}+{\left(p-q\right)}^{2}t^{2}>{\left(p+q\right)}^{2}f^{2},$$

it is easy to show that

$$x^{*}=\frac{-2qf+\left(p-q\right)t+\sqrt{4pqf^{2}+{\left(p-q\right)}^{2}t^{2}}}{2\left(p-q\right)}<\frac{t+f}{2}.$$

For *p* < *q* and *t* > *f* the function $h\left(x\right)=g\left(x\right)-x$ is a convex function in the interval *M*. Indeed, its second-degree derivative is equal to 𝑔″(*x*) and positive in this interval:

$$h^{\prime \prime }\left(x\right)=g^{\prime \prime }\left(x\right)=\frac{-2pq\left(p-q\right)\left(t^{2}-f^{2}\right)}{{\left(\left(p-q\right)x+q\left(t+f\right)\right)}^{3}}>0,$$

because for *x* ∈ *M*, we have:

$$\left(p-q\right)x+q\left(t+f\right)\geq \left(p-q\right)\left(t+f\right)+q\left(t+f\right)=p\left(t+f\right)>0.$$

This means that a maximum of this function is reached at one of the ends of a considered range. Therefore for the final part of the interval *M*: $\frac{t+f}{2}\leq x_{i}\leq t+f$ we can estimate the value $x_{i+1}-x_{i}$ from above by $\rho_{-}=\max\left\{g\left(\frac{t+f}{2}\right)-\frac{t+f}{2},g\left(t+f\right)-\left(t+f\right)\right\}<0$, i.e.,

$$x_{i+1}-x_{i}\leq \rho_{-}=\max\left\{g\left(\frac{t+f}{2}\right)-\frac{t+f}{2},g\left(t+f\right)-\left(t+f\right)\right\}<0$$

$$x_{i+1}-x_{i}=\frac{-\left(p-q\right)x_{i}^{2}+\left(\left(p-q\right)t-2qf\right)x_{i}+q\left(t+f\right)f}{\left(p-q\right)x_{i}+q\left(t+f\right)}\leq g\left(\frac{t+f}{2}\right)-\frac{t+f}{2}\;=\frac{\left(p-q\right)\left(t-f\right)}{2\left(p+q\right)}=\frac{p-q}{p+q}\cdot \frac{t-f}{2}<0.$$

$$x_{i+1}-x_{i}=\frac{-\left(p-q\right)x_{i}^{2}+\left(\left(p-q\right)t-2qf\right)x_{i}+q\left(t+f\right)f}{\left(p-q\right)x_{i}+q\left(t+f\right)}\leq g\left(t+f\right)-\left(t+f\right)\;=\frac{-pt-pf+qt+qf+pt-qt-2qf+qf}{p}=-f<0.$$

Hence,

$$x_{i+1}-x_{i}\leq \rho_{-}=\max\left\{-f,\frac{p-q}{p+q}\cdot \frac{t-f}{2}\right\}<0,x_{i+1}<x_{i}.$$

So for $\frac{t+f}{2}\leq x_{i}\leq t+f$, the sequence ($x_{i}$)_*i*=0…∞_ is strictly decreasing and while $x_{i}\geq \frac{t+f}{2}$:

$$x_{i+1}\leq x_{i}+\rho_{-}=x_{i}-\left|\rho_{-}\right|<x_{i}.$$

Thus, if $\frac{t+f}{2}\leq x_{0}\leq t+f$, then $x_{1}\leq x_{0}+\rho_{-}\leq t+f+\rho_{-}=t+f-\left|\rho_{-}\right|<t+f$. Similarly, if $\frac{t+f}{2}\leq x_{1}\leq t+f+\rho_{-}$, then $x_{2}\leq x_{1}+\rho_{-}\leq t+f+2\rho_{-}=t+f-2\left|\rho_{-}\right|$. Generalizing, if $\frac{t+f}{2}\leq x_{i}\leq t+f+i\rho_{-}$, then $x_{i+1}\leq x_{i}+\rho_{-}\leq t+f+\left(i+1\right)\rho_{-}=t+f-\left(i+1\right)\left|\rho_{-}\right|$. This means that if for *i* ≤ *n*, where $n=\lceil \frac{t+f}{2\left|\rho_{-}\right|}\rceil \geq \frac{t+f}{2\left|\rho_{-}\right|}$ (⌈⌉ denotes a ceiling function) all $x_{i}\geq \frac{t+f}{2}$, then $x_{n}\leq t+f-\left(n+1\right)\left|\rho_{-}\right|\leq t+f-\left(\frac{t+f}{2\left|\rho_{-}\right|}+1\right)\left|\rho_{-}\right|=\frac{t+f}{2}-\left|\rho_{-}\right|<\frac{t+f}{2}$. In other words, among the *n* + 1 first elements of the sequence ($x_{i}$)_*i*=0…∞_, there must appear an element less than $\frac{t+f}{2}$. So some element of the sequence ($x_{i}$)_*i*=0…∞_ lies below $\frac{t+f}{2}$. Let us denote the first such element with the index *j*.

Now assuming *t* > *f*, we can write for $x_{i}<\frac{t+f}{2}$:

$$\left|x_{i+2}-x_{i+1}\right|=\frac{pq\left(t-f\right)\left(t+f\right)}{\left(p-q\right)\left(p+q\right)tx_{i}+q\left(pf+qt\right)\left(t+f\right)}\left|x_{i+1}-x_{i}\right|\;<\frac{pq\left(t-f\right)\left(t+f\right)}{\left(p-q\right)\left(p+q\right)t\frac{1}{2}\left(t+f\right)+q\left(pf+qt\right)\left(t+f\right)}\left|x_{i+1}-x_{i}\right|\;=\frac{2pq\left(t-f\right)}{p^{2}t+q^{2}t+2pqf}\left|x_{i+1}-x_{i}\right|.$$

If *t* > *f* and $x_{i}<\frac{1}{2}\left(t+f\right)$, then

$$k_{\mp }=\frac{2pq\left(t-f\right)}{p^{2}t+q^{2}t+2pqf}<1$$

because *p*²*t* + *q*²*t* + 2*pqf* > 2*pq*(*t* − *f*) > 0, since (*p* − *q*)²*t* > −4*pqf*.

It means that for *p* < *q* and *t* > *f*, the sequence ($x_{i}$)_*i*=0…∞_ is a Cauchy sequence in the interval *M*, i.e., $\left|x_{i+2}-x_{i+1}\right|<k_{\mp }\left|x_{i+1}-x_{i}\right|$ for *i* = *j*…∞ and some $k_{\mp }<1$, and it is convergent.

To recapitulate, the paper shows that for *f* ≠ 0 and *t* ≠ 0, the sequence ($x_{i}$)_*i*=0…∞_ starting from any $x_{0}$ ∈ *M* is convergent to a single fixed point in *M*, but for *f* = 0 or *t* = 0, it is not true. For *t* = 0, the sequence (*x_i_*)_*i*=0…∞_ generally oscillates around a single fixed point and is convergent only when it is constant and starts exactly from the fixed point. For *f* = 0 and *p* ≠ *q*, the sequence ($x_{i}$)_*i*=0…∞_ is always convergent to one of the specific ends of the interval *M*, while the other end is also a fixed point but only reachable by the constant sequence starting exactly from this point. For *f* = 0 and *p* = *q*, every point of the interval *M* is a fixed point, and every sequence ($x_{i}$)_*i*=0…∞_ starting from any $x_{0}$ ∈ *M* is always constant and converges to this point.

If the starting point $x_{0}$ lies outside the interval *M* (but is greater than zero), its first iteration $x_{1}$ will fall into the interval *M*, so the above conclusions are also valid for $x_{0}\geq 0$. Identical conclusions also apply to the sequence ($y_{i}$)_*i*=0…∞_.

## 7. Discussion

It is of special interest to wireless multi-hop IoT networks to see whether the process of convergence can be negatively affected by the dynamics of the transport layer. Specifically, phenomena such as delays and repetitions of opinions, as well as the disappearance of nodes, are of certain concern.

The convergence itself seems to be resilient to the majority of such negative cases. Neither the algorithm nor its convergence makes any assumption about the timeliness or order of appearance of opinions. It also makes no assumption about the continuity of the existence of nodes so that it is resilient to the appearance and disappearance of nodes. The eventuality of convergence implies that delays in the delivery of opinions should make no impact on the process of convergence, barring the decrease in the speed of this process.

The algorithm itself relies on benevolent nodes having stable identities and sharing the approximately correct clock. It shares those assumptions with several reputation-based schemes. Consequently, attacks on identity may have an impact on the calculation of reputation. The assurance regarding the identity can be achieved, e.g., by using cryptography (see, e.g., the work of [30]). Note, however, that those attacks should not have an impact on the convergence itself, only on the value the algorithm converges to.

## 8. Conclusions

This paper presents the analysis and the proof of eventual convergence for the EWMA algorithm, as used in IoT sensor networks. Such an algorithm can be used in over-provisioned (“dense”) sensor networks to decrease the uncertainty of measurements reported by the networks, even if low-cost sensors are used, and no reference nodes are present.

The novelty of this paper lies in the demonstration of the proof of the convergence of the EWMA algorithm, as it can be used in IoT sensor networks. The convergence of the EWMA algorithm has been studied before, but those results are not directly applicable to the way this algorithm can be used in IoT sensor networks. For uses cases similar to the IoT networks, the convergence is usually assumed and demonstrated through the simulation. The demonstration of the mathematical proof means that the algorithm can be safely used in situations where the assurance of convergence is required.

The paper starts by describing the EWMA algorithm in a way applicable to IoT sensor networks. Then the UOG is introduced as a model of random graphs that well represents IoT sensor networks while allowing for formalization. It is the formalization provided by the UOG that bridges the application and the mathematics of the EWMA. The preliminary analysis of the convergence of the EWMA for the UOG is then conducted. From there, the mathematical proof follows. It demonstrates that, apart from some inapplicable cases, the algorithm assures eventual convergence.

The EWMA algorithm, as described in this paper, does not rely on any particular configuration of the transport layer. The sensitivity of the convergence to the variability of the IoT network is currently being studied. It is already visible that the eventuality of the convergence makes it resilient to negative aspects of the dynamics of wireless multi-hop networks, such as delays, repetitions, disappearance, and non-delivery. The suggested implementation does not require a significant increase in the computing power of the nodes, and its impact on the required bandwidth can be contained as well. Models such as the work of [31] can be used to further optimize the energy consumption, if required.

The algorithm assumes that all the nodes are benevolent, even though some of them may be faulty or of lower quality. The behavior of the algorithm suggests that in case of an adversary attack where, e.g., malicious nodes are added to the network or benevolent ones became malicious, the algorithm may still deliver convergence, but it may not converge on the correct vector of values. The behavior of the algorithm in such situations is currently being studied.

## Data Availability

Not applicable.

## Glossary

**Abbreviation**	**Meaning**
AEWMA	Adaptative EWMA, see the work of [17]
EWMA	Exponentially Weighted Moving Average
IoT	Internet of Things
PEWMA	Probabilistic EWMA, see the work of [16]
SMA	Simple Moving Average
UOG	Uniform Opinion Graph
**Symbol**	**Meaning**
*a, b,..*	the sensor within the sensor network
*τ*	moment in time
*w*	reputation
*u*	level of trust
*conf*	level of confidence of one sensor into the other
*c*	level of self-confidence
*f_d_*	decay function
*λ*	decay constant used by the *f_d_*
*x_i_*	reputation of a representative trustworthy node at the iteration *i*
*y_i_*	reputation of a representative faulty node at the iteration *i*
*p*	the number of trustworthy nodes that provide opinions about a given node
*q*	the number of faulty nodes that provide opinions about a given node
*t*	the level of trust reported by the compatible node
*i*	the level of trust reported by the incompatible node
*(x**, *y**)	the convergence vector
*M*	the convergence interval
*m*_-_, *m*_+_	bracketing values of *M*
*g*(*x*)	substitute function replacing vectors with a sequence
*x*_-_*, *x*_+_*	fixed points of *g(x)*
*α*	substitute sequence used to express *g(x)*

## References

[1] O’Donovan J., Golbeck J. (2009). Capturing Trust in Social Web Applications. Computing with Social Trust.

[2] Cofta P., Karatzas K., Orłowski C. (2021). A Conceptual Model of Measurement Uncertainty in IoT Sensor Networks. Sensors.

[3] Yun J., Woo J. (2021). IoT-Enabled Particulate Matter Monitoring and Forecasting Method Based on Cluster Analysis. IEEE Internet Things J..

[4] Syed A.S., Sierra-Sosa D., Kumar A., Elmaghraby A. (2021). IoT in Smart Cities: A Survey of Technologies, Practices and Challenges. Smart Cities.

[5] D’Amico G., L’Abbate P., Liao W., Yigitcanlar T., Ioppolo G. (2020). Understanding Sensor Cities: Insights from Technology Giant Company Driven Smart Urbanism Practices. Sensors.

[6] Ma Z., Liu L., Meng W. DCONST: Detection of Multiple-Mix-Attack Malicious Nodes Using Consensus-Based Trust in IoT Networks. Proceedings of the Australasian Conference on Information Security and Privacy (ACISP 2020).

[7] Djedjig N., Tandjaoui D., Romdhani I., Medjek F., Maleh Y., Ezzati A., Belaissaoui M. (2018). Trust Management in the Internet of Things. Security and Privacy in Smart Sensor Networks.

[8] Azzedin F., Ghaleb M. (2019). Internet-of-Things and Information Fusion: Trust Perspective Survey. Sensors.

[9] Chen D., Chang G., Sun D., Li J., Jia J., Wang X. (2011). TRM-IoT: A Trust Management Model Based on Fuzzy Reputation for Internet of Things. ComSIS.

[10] Awan K.A., Din I.U., Almogren A., Almajed H., Mohiuddin I., Guizani M. (2020). NeuroTrust-Artificial Neural Network-based Intelligent Trust Management Mechanism for Large-Scale Internet of Medical Things. IEEE Internet Things J..

[11] Jøsang A., Ismail R., Boyd C. (2007). A survey of trust and reputation systems for online service provision. Decis. Support Syst..

[12] Chang E., Dillon T., Hussain F. (2006). Trust and Reputation for Service-Oriented Environments. Technologies for Building Business Intelligence and Consumer Confidence.

[13] Sukparungsee S., Areepong Y., Taboran R. (2020). Exponentially weighted moving average—Moving average charts for monitoring the process mean. PLoS ONE.

[14] Yu J., Kim S.B., Bai J., Han S.W. (2020). Comparative Study on Exponentially Weighted Moving Average Approaches for the Self-Starting Forecasting. Appl. Sci..

[15] Cofta P., Orłowski C., Lebiedź J. (2020). Trust-Based Model for the Assessment of the Uncertainty of Measurements in Hybrid IoT Networks. Sensors.

[16] Renshaw J. (2019). Anomaly Detection Using aws iot and aws Lambda. https://aws.amazon.com/blogs/iot/anomaly-detection-using-aws-iot-and-aws-lambda/.

[17] Capizzi G., Masarotto G. (2003). An Adaptive Exponentially Weighted Moving Average Control Chart. Technometrics.

[18] Eckner A. (2015). Algorithms for Unevenly Spaced Time Series: Moving Averages and Other Rolling Operators. https://www.semanticscholar.org/paper/Algorithms-for-Unevenly-Spaced-Time-Series-%3A-Moving-Eckner/882e93570eae184ae737bf0344cb50a2925e353d.

[19] Vinod H.D. (2006). Maximum entropy ensembles for time series inference in economics. J. Asian Econ..

[20] Steiner S.H. (1999). Exponentially Weighted Moving Average Control Charts with Time-Varying Control Limits and Fast Initial Response. J. Qual. Technol..

[21] Hosseini S.S., Noorossana R. (2018). Performance evaluation of EWMA and CUSUM control charts to detect anomalies in social networks using average and standard deviation of degree measures. Qual. Reliab. Eng..

[22] West A.G., Lee I., Kannan S., Sokolsky O., Zheng Y. (2010). An Evaluation Framework for Reputation Management Systems. Trust Modeling and Management in Digital Environments: From Social Concept to System Development.

[23] Hillam K.L., Thron W.J. (1965). A general convergence criterion for continued fractions K (an/bn). Proc. Am. Math. Soc..

[24] Mandell M., Magnus A. (1970). On convergence of sequences of linear fractional transformations. Math. Z..

[25] Mui L. (2002). Computational Models of Trust and Reputation: Agents, Evolutionary Games, and Social Networks. Ph.D. Thesis.

[26] Carbo J., Molina J., Davila J. (2003). Trust Management Through Fuzzy Reputation. Int. J. Coop. Inf. Syst..

[27] Akbani R., Korkmaz T., Raju G.V.S. A Machine Learning Based Reputation System for Defending Against Malicious Node Behavior. Proceedings of the IEEE GLOBECOM 2008—2008 IEEE Global Telecommunications Conference.

[28] Alnumay W., Ghosh U., Chatterjee P. (2019). A Trust-Based Predictive Model for Mobile Ad Hoc Network in Internet of Things. Sensors.

[29] Cofta P. (2007). Trust, Complexity and Control: Confidence in a Convergent World.

[30] Verri Lucca A., Mariano Sborz G.A., Leithardt V.R.Q., Beko M., Albenes Zeferino C., Parreira W.D. (2021). A Review of Techniques for Implementing Elliptic Curve Point Multiplication on Hardware. J. Sens. Actuator Netw..

[31] dos Anjos J.C.S., Gross J.L.G., Matteussi K.J., González G.V., Leithardt V.R.Q., Geyer C.F.R. (2021). An Algorithm to Minimize Energy Consumption and Elapsed Time for IoT Workloads in a Hybrid Architecture. Sensors.

